# Effect of very low-calorie ketogenic diet in combination with omega-3 on inflammation, satiety hormones, body composition, and metabolic markers. A pilot study in class I obese subjects

**DOI:** 10.1007/s12020-021-02860-5

**Published:** 2021-09-16

**Authors:** Mariangela Rondanelli, Simone Perna, Zahra Ilyas, Gabriella Peroni, Philip Bazire, Ignacio Sajuox, Roberto Maugeri, Mara Nichetti, Clara Gasparri

**Affiliations:** 1 IRCCS Mondino Foundation, Pavia, 27100 Italy; 2 Department of Public Health, Experimental and Forensic Medicine, University of Pavia, Pavia, 27100 Italy; 3 Department of Biology, College of Science, University of Bahrain, Sakhir, 32038 Bahrain; 4 Endocrinology and Nutrition Unit, Azienda di Servizi alla Persona “Istituto Santa Margherita”, University of Pavia, Pavia, 27100 Italy; 5 Medical Department, PronoKal Group, London, UK; 6 Scientific Officer, PronoKal Group, Barcellona, 08009 Spain; 7 Medical Director, PronoKal Group, Savigliano, 12038 Italy

**Keywords:** Ketogenic diet, Obesity, Visceral adipose tissue, Omega-3 fatty acids, Weight loss

## Abstract

**Purpose:**

This study aims to evaluate the effects of a VLCKD combined with omega-3 supplementation (VLCKD diet only lasted for some weeks, and it was followed by a non-ketogenic LCD for the rest of the study period) on body composition, visceral fat, satiety hormones, inflammatory and metabolic markers.

**Methods:**

It has been performed a pilot open label study lasted 90 days, in a cohort of 12 women with class I obesity aged 18 to 65 years. Data on body composition (evaluated by Dual X-Ray Absorptiometry—DXA), visceral fat, satiety hormones, inflammatory and metabolic markers were recorded.

**Results:**

This study showed a body weight reduction mean difference over time of −13.7 kg and the waist circumference mean difference decrease of −13.3 cm. Also, the fat mass (FM) decreased—9.1 kg and visceral adipose tissue (VAT)—0.41 kg. No effects on fat-free mass (FFM) have been reported. Improvements were observed in the satiety hormones, with increased ghrelin and decreased leptin, and also in the metabolic profiles.

**Conclusions:**

A VLCKD combined with omega-3 supplementation appears to be an effective strategy for promoting an high loss of FM with preservation of FFM in patients with class I obesity.

## Introduction

To date, the best noninvasive strategies for weight loss have been based on dietary interventions and behavioral change [1, 2]. Lifestyle modification should be the foundation of any treatment plan that aims to decrease calorie intake and increase energy expenditure. Diet- and exercise-induced weight loss have been examined in numerous studies [3].

The Very Low-Calorie Ketogenic Diet (VLCKD) is an effective and used tool for weight loss in people with obesity. VLCKDs are characterized by a very low carbohydrate content (<20 g/day), 1–1.5 g protein/kg ideal body weight, 15–30 g fat/day, and a daily energy intake of 500 to 800 calories [4]. VLCKD could lead to rapid weight loss the induction of satiety and the preservation of muscle mass [5] alone recent in vivo study shows that [6], using a very low carbohydrate, low-protein, high-fat ketogenic diet, observed a reduction or even complete suppression of the expression of inflammatory cytokines and chemokines, and of the production of reactive oxygen species.

The VLCKD produced a decrease in visceral adipose tissue (VAT), reducing adiposity and improving blood biochemistry parameters, thus leading to a reduction in cardiovascular risk factors [7]. In addition, VLCKDs decreases BMI, waist circumference, intermuscular adipose tissue, total body fat, HDL-cholesterol (HDL-C), LDL-C, C-reactive protein (CRP), and blood glucose levels throught the modulation of SOD1 expression.

A recent study showed that a ketogenic diet and fish-oil supplementation improve health, inflammation status [8]. And produced a significant loss of body weight, body fat. blood glucose, total cholesterol, and LDL-C levels and decrease on inflammatory cytokines (interleukin [IL]-1β, IL-6, TNF-α)

The combined effects of a low-carbohydrate, high-protein diet plus omega-3 PUFA supplementation is not fully understood. In particular, Omega-3 polyunsaturated fatty acids (PUFAs) have also been shown to reduce hepatic steatosis and reduce the effects of visceral adipose tissue by influencing lipid metabolism and insulin sensitivity [9].

This combination between diet and omega 3 could improve insulin action may especially be associated with long-chain-polyunsaturated omega-3 fatty acids (LCPUFAn-3) in the structural lipids of skeletal muscle and among the LCPUFAn-3, docosahexaenoic acid (DHA) may be the most important fatty acid in this context [10].

This study aims to evaluate the effects of a VLCKD combined with omega-3 supplementation (VLCKD diet only lasted for some weeks, and it was followed by a non-ketogenic LCD for the rest of the study period) on body composition, visceral fat, satiety hormones, inflammatory and metabolic markers.

## Methods

### Study design and participants

This was an uncontrolled, single-center, open label pilot clinical study. Participants were recruited at the Endocrinology and Nutrition Unit of the Azienda di Servizi alla Persona, Istituto Santa Margherita, University of Pavia (27100 Pavia, Italy) Participants received no monetary incentive, and all provided written informed consent. The study was performed following approval by the Ethics Committee of the University of Pavia (Reg. no. 9309/14122018). All procedures involving human participants were performed in accordance with the ethical standards of the Ethics Committee of the University of Pavia and with the 1964 Helsinki Declaration and its later amendments or comparable ethical standards.

### Inclusion and exclusion criteria

Adult men and women aged 18–65 years, with a body mass index (BMI) between 30 and 35 kg/m^2^, wishing to undergo weight loss, and with a history of failed dietary attempts, were eligible to participate. The following exclusion criteria were applied: administration of omega-3 fatty acids, acetylsalicylic acid, nonsteroidal anti-inflammatory drugs, or corticosteroids in the month prior to study entry; pregnancy or breastfeeding; blood disorders (coagulation disorders, or therapy with coumadin anticoagulants); a history of cancer; type 1 or type 2 diabetes mellitus; and immune (rheumatoid arthritis, lupus, etc.) or inflammatory (ulcerative colitis, Crohn’s disease, etc.) diseases likely to modify biological markers of inflammation. Failure to comply with the guidelines and non-attendance at follow-up visits led to exclusion.

### Dietary intervention

The intervention period was of 3 months. Patients initially received a very low calorie ketogenic diet (KD), based on an intake of protein-food preparations plus supplementation with vitamins, minerals, and omega-3 fatty acids (DHA and EPA). This was followed by a low-calorie diet (LCD). A commercial weight-loss program was used for the KD (Pronokal^®^ method, Barcelona, Spain); this program provides high-biological-value protein preparations and natural foods. Each protein preparation contains 15 g protein, 4 g carbohydrate, 3 g fat, and 50 mg DHA, with an energy content of 90–100 kcal. This program has three stages: active weight loss, dietary re-education, and maintenance (Table 1). The active stage consists of a very low calorie (600–800 kcal/day), low carbohydrate (20–50 g/day from vegetables), and low-fat (10 g/day of olive oil) diet. The intake of high-biological-value protein is adjusted to between 0.8 and 1.2 g per kg ideal body weight per day, to cover minimum requirements and to prevent the loss of lean mass. The active stage is divided into three steps. In step 1, the patients eat high-biological-value protein preparations five times a day, plus low-glycemic-index vegetables. In step 2, one of the protein servings is substituted by a natural protein food (e.g., meat or fish), either at lunch or at dinner. In step 3, a second protein preparation is substituted by a natural protein food. Supplementation with vitamins, minerals (including potassium, sodium, magnesium and calcium), and omega-3 fatty acids is provided in accordance with international recommendations throughout the ketogenic stage of the diet [11]. This active stage is continued until the patient has lost about 80% of the target weight loss. The duration of the ketogenic stage of the program therefore varies depending on the individual rate of weight loss and on the weight loss target, but in all our patients was between 30 and 45 days. The ketosis stage is terminated by the physician after adequate weight loss, and patients move into the low-calorie, dietary re-education stage. The different food groups are progressively reintroduced during this stage, and patients participate in a dietary re-education program designed to favor long-term weight stability. The low calorie diet period is continued until the patient has lost the remaining 20% of the target weight loss. The maintenance stage consists of an eating plan balanced in carbohydrates, protein, and fat, and the promotion of a healthy lifestyle. Energy content of the maintenance diet varies between 1500 and 2000 kcal/day, depending on the individual, aiming to maintain the weight loss.

**Table 1:** The timeline of dietary intervention

80% of the target weight loss			20% of the target weight loss		Manteinance
Weight loss					New lifestyle
Stage 1	Stage 2	Stage 3	Stage 4	Stage 5	
50% of 80% of the target weight loss	25% of 80% of the target weight loss	25% of 80% of the target weight loss	50% of 20% of the target weight loss	50% of 20% of the target weight loss	
Very low calorie ketogenic diet			Low calorie diet		Balanced diet
(630–700 kcal/day)			(800–1500 kcal/day)		(1500–2000 kcal/day)
Omega Balance: 700 DHA/500 EPA			DHA Vita: 500 DHA/100 EPA		DHA Vita: 500 DHA/100 EPA

### Lifestyle intervention

Physical activity and psychological support are also included as an integral part of the program. During the ketosis stage, patients are required to perform a regimen of full-body resistance exercises at least three times a week; from the 4th stage, aerobic exercise (walking or cycling) of at least 1 h twice a week is added. For the present study, a telephone support plan was set up, and a phone number was provided to all participants to address doubts.

### Compliance

Adherence to the diet and exercise recommendations was determined through self-reporting for exercise, food records, and measurement of blood ketone levels with a finger-prick test at all hospital visits. Capillary blood ketone measurement was performed using the FreeStyle Optium system, with the FreeStyle Optium Neo ketone control solution and reagents and FreeStyle Optium β-ketone test strips.

### Follow up

The study included a total of two visits (at baseline and at 3 months) for the body composition assessment and for measurement of all biochemical markers.

### Body composition analysis

#### Body composition

Body composition was measured by total body imaging. Data were acquired using the Lunar Dual X-Ray Absorbimetry (DXA) (GE Healthcare, Madison, WI, USA), and analyzed using enCORE software version 13.2. The scanner was calibrated daily against the calibration block supplied by the manufacturer. No hardware or software changes were made during the course of the trial. Subjects were scanned using standard imaging and positioning protocols, while wearing only light clothing. For the current study we used bone mineral density, lean body mass, and fat mass (FM) values, which are determined directly by the GE Lunar Body Composition Software (GE Healthcare). Some derivative values, such as bone mineral content, regional lean mass, arm lean mass, fat-free mass (FFM), FM percentage (FM%), and android and gynoid fat percentages, were also calculated. The android-to-gynoid ratio was automatically generated and analyzed using enCORE software, version 13.6. For measuring android fat, a region of interest was automatically defined with a caudal limit at the top of the iliac crest, and a cephalad limit at 20% of the distance from the top of the iliac crest to the base of the skull. Body composition was also measured by bioelectrical impedance analysis (BIA).

#### Visceral fat mass

Visceral fat was calculated using a software (Core Scan, GE Healthcare), which has been validated against computed tomography in a patient population with a wide range of BMIs [12, 13]. Visceral fat mass data from DXA were transformed into adipose tissue volume using a constant correction factor (0.94 g/cm^3^).

### Biochemical markers

Fasting blood samples were drawn from subjects immediately prior to initiating the study and after 3 months. Hemoglobin A1c, fasting blood glucose, fasting lipid profile (LDL-C and HDL-C), and serum albumin were measured using standard automated laboratory techniques (Beckman Coulter LX 20, Fullerton, CA, USA; Tosoh A1C 2.2 Analyzer, Tokyo, Japan). CRP was measured in triplicate using the Human C-Reactive Protein ELISA Kit (ADI, San Antonio, TX, USA). HOMA-IR was calculated as: (fasting glycemia x fasting insulin)/405, with a range of normality from 0.23 to 2.5 [14].

Leptin and insulin were measured with radioimmunoassay kits from LINCO Research. The intra-assay coefficient of variation was between 4.1 and 8.1% for leptin and between 4.2 and 9.3% for insulin. Acyl ghrelin and des-acyl ghrelin were measured using a double-antibody sandwich assay by Thorner Laboratory (University of Virginia, Charlottesville, VA, USA), which prevents the detection of inactive peptide fragments [15].

### Statistical analysis

No parametric distribution was assumed for any of the outcome variables because of the small sample size.

The observed means were used to better understand outcome variation at each follow-up. In addition, the mean ± SD (for continuous variables) and Fisher’s exact test (for categorical variables) were used to compare baseline characteristics and primary and secondary outcome measures between the different study visits.

Spearman’s correlation was used to assess the association between pre/post intervention changes (delta) and to analyze the effect of leptin and ghrelin on body composition and biochemical markers. Statistical power was 80%.

Only subjects who completed the study and had both baseline and final measurements were included in the analysis. Because this was a pilot study, no power calculations were performed, and all findings need to be confirmed in a larger clinical trial. All statistical analyses were performed using SPSS software, version 25 (IBM, Armonk, NY, USA).

## Results

Fifteen individuals agreed to participate. Three were excluded from the analysis: one for non-compliance, one dropped out because the capsules were too large, and one moved away from the area and was lost to follow-up. Thus, twelve participants (12 women and 3 men), aged 50 ± 12.27 were included in the analyses.

The baseline characteristics are shown in Table 2.

**Table 2:** Parameters evaluated at baseline

Variable	Median	IQR
Age (years)	56.5	19.75
Weight (kg)	94.70	19.58
Height (cm)	170.75	0.44
BMI (kg/m^2^)	33.84	2.72
Waist circumference (cm)	109.00	16.25
Leptin (ng/mL)	48.00	38.00
Ghrelin (pg/mL)	11.80	12.79
CRP (mg/L)	0.37	0.44
Glucose (mg/dL)	85.00	8.00
Insulin (mcU/mL)	14.45	7.00
HOMA-IR index	2.83	1.72
Uric acid (mg/dL)	5.15	1.20
BUN (mg/dL)	33.00	9.00
Creatinine (mg/dL)	0.86	0.16
eGFR (mL/min)	84.50	18.00
Total cholesterol (mg/dL)	183.00	41.00
Triglycerides (mg/dL)	98.00	49.00
AST (U/L)	20.00	6.00
ALT (U/L)	18.50	14.00
GGT (U/L)	17.50	27.00

*ALT* alanine aminotransferase, *AST* aspartate aminotransferase, *BMI* body mass index, *BUN* blood urea nitrogen, *CRP* C-reactive protein, *eGFR* estimated glomerular filtration rate, *GGT* gamma glutamyl transferase, *HOMA-IR* h omeostasis model assessment of insulin resistance

Table 3 shows the pre/post (90 days) difference in means. No statistically significant improvement was recorded in biochemical marker values, except for ghrelin, which presented an increase of 0.16 pg/mL (*P* = 0.001)

**Table 3:** Biochemical markers: mean differences pre/post intervention at 90 days

Variable	Pre	Post	W	*P*
Leptin (ng/mL)	40.09	10.70	0.918	0.338
Ghrelin (pg/mL)	16	30.05	0.706	0.001*
CRP (mg/L)	0.42	0.26	0.909	0.270
Glucose (mg/dL)	85.58	81.50	0.980	0.966
Insulin (mcU/mL)	14.98	6.49	0.932	0.468
HOMA-IR index	3.18	1.31	0.944	0.602
Uric acid (mg/dL)	4.84	5.53	0.876	0.118
BUN (mg/dL)	34.0	35.1	0.875	0.115
Creatinine (mg/dL)	0.82	0.86	0.933	0.480
eGFR (mL/min)	88.67	88.40	0.906	0.252
Total Cholesterol (mg/dL)	190.92	170.20	0.942	0.578
Triglycerides (mg/dL)	106.25	78.30	0.648	0.284
AST (U/L)	19.42	18.30	0.849	0.066
ALT (U/L)	20.17	23.33	0.794	0.061

*ALT* alanine aminotransferase, *AST* aspartate aminotransferase, *BUN* blood urea nitrogen, *CRP* C-reactive protein, *eGFR* estimated glomerular filtration rate, *GGT* gamma glutamyl transferase, HOMA-IR homeostasis model assessment of insulin resistance, *W* Wilcoxon Signed Rank Test

**Fig. 1:**
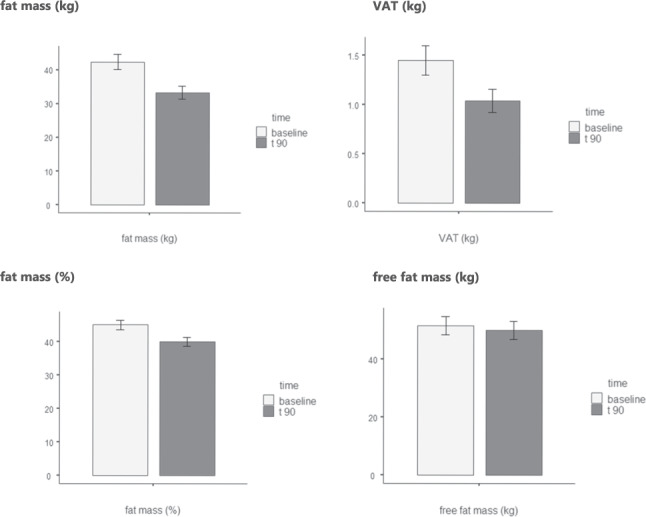
Body composition: difference in means pre/post intervention (90 days) of fat mass (in kg and %), visceral adipose tissue (VAT, in kg) and fat free mass (in kg). The administration of VLCKD reduced FM by 9.1 kg (from 42.4 to 33.3 kg) and VAT by 0.41 kg (from 1.45 to 1.04 kg). The FM% decreased from 45.1% to 40.1%. The change in the FFM was not significant (decrease from 51.6 to 50.0 kg)

Figure 1 shows the changes in body composition parameters from baseline to the end of intervention with the VLCKD. The administration of this VLCKD over a period of three months reduced FM by 9.1 kg (from 42.4 to 33.3 kg) and VAT by 0.41 kg (from 1.45 to 1.04 kg). The FM% decreased from 45.1% to 40.1%. The change in the FFM was not significant (decrease from 51.6 to 50.0 kg).

Table 4 shows the changes in anthropometric parameters from baseline to the end of intervention with the VLCKD. The administration of the VLCKD for three months reduced body weight by 13.7 kg (from 98.9 to 85.2 kg), with the consequent reduction in the BMI from 33.7 kg/m^2^ (class I obesity) to 29.0 kg/m^2^ (overweight). Waist circumference decreased by 13.3 cm, from 110.2 to 96.9 cm.

**Table 4:** Anthropometric parameters pre and post intervention

Variable	Mean value at baseline ± ds	Mean value at the end of the treatment ± ds	Difference in means	Percentage change from baseline
Body weight (kg)	96.63 ± 16.56	85.19 ± 15.3	−11.44	−11.84%
Body mass index (kg/m^2^)	33.46 ± 1.63	28.99 ± 1.52	−4.47	−13.36%
Waist circumference (cm)	109.00 ± 9.99	96.90 ± 7.92	−12.10	−11.10%

Table 5 shows the monitoring of blood ketones during the VLCKD phase. The ketone values remained constant within the desirable range of 0.6–1.5 mmol/L in ketosis.

**Table 5:** Blood ketones monitoring during the VLCKD phase

Variable	Mean value ± ds day 15	Mean value ± ds day 30	Mean value ± ds day 45
Blood ketones (mmol/L)	0.72 ± 0.43	0.81 ± 0.59	0.86 ± 0.58

Table 6 shows the Spearman’s correlation between pairs of variables considered.

**Table 6:** Spearman’s correlation between pairs of variables considered

Spearman’s correlations								
Variable		Δghrelin	Δ leptin	Δ ketones	Δ crp	Δ insuline	Δ cholesterol	Δ fat mass
1. Δghrelin	(r)	—						
2. Δ leptin	(r)	0.41	—					
3. Δ ketones	(r)	0.05	0.55	—				
4. Δ crp	(r)	0.10	0.15	0.82	—			
5. Δ insulin	(r)	−0.10	0.05	0.56	0.30	—		
6. Δ cholesterol	(r)	0.50	−0.21	0.36	0.70	0.30	—	
7. Δ fat mass	(r)	−0.10	−0.87	−0.56	−0.30	0.20	0.30	—

*CRP* c-reactive protein

*p < 0.05, **p < 0.01, ***p < 0.001

## Discussion

On the basis of our results, the administration of a VLCKD for a period of 3 months would appear to be an effective strategy for promoting weight loss in patients with class I obesity. Our pilot study has demonstrated that patients on a VLCKD achieve good weight loss (13.8% weight loss from baseline) in a 3-month intervention, with a 13.9% fall in BMI, which meant that these patients were no longer obese and had moved into the overweight category. Importantly, weight was lost principally as a loss of FM and of VAT, whereas the FFM remained stable.

The decrease of 9.1 kg in FM found in our study supports a previous study by Gomez-Arbelaez et al. [16]. Those authors used a commercial VLCKD program (the PNK Method, which includes lifestyle and behavior modification) in a 4-month intervention, achieving significant weight loss (20.2 kg), mainly targeting FM (decrease of 16.5 kg) and VAT. In that study, there was a slight initial loss of FFM, but this subsequently showed partial recovery, and was considered to be due to transient changes in body water balance related to the intense diuresis that occurs in the early phase of any VLCKD.

Importantly, our study highlighted the excellent ability of the VLCKD to preserve the FFM. We detected no statistically significant loss of FFM. This result also coincides with the aforementioned study by Gomez-Arbelaez, in which the loss of FFM of around 1 kg over the 4-month study represented only 5% of the total weight lost. The observation that the VLCKD produced a marked reduction in FM while preserving muscle mass, was reinforced by the maintenance of muscle strength during the course of the diet [16].

The overall effect of VLCKDs on body composition was investigated by Merra et al., who compared a VLCD with a VLCKD in which 50% of protein intake came from an amino acid supplement [17]. Those authors reported better preservation of lean body mass during weight loss with the VLCKD, protecting against diet-induced sarcopenia [17]. KD appear to protect against the loss of muscle mass seen with aging or during LCDs; this effect of KDs is attributed to the maintenance of muscle mass rather than a net hypertrophic effect [18]. The concept was confirmed by Vargas Molina et al., who investigated the effect of a KD on body composition and strength in a sample of trained women participating in an 8-week program of resistance training (RT) [19]. The KD led to a decrease in fat mass and maintenance of fat free mass after 8 weeks of RT in trained women, but was suboptimal for increasing FFM [19]. The interpretation of study results on FFM can be problematic. One confounding factor might be the amount of daily protein intake expressed as grams of protein per kilogram body mass and the fitness level of subjects (sedentary or active/athletic) [18].

Another relevant finding of the present pilot study is the 12% reduction in waist circumference, reflecting the decrease in VAT of around 400 g. The improvement in VAT indicates a reduction in abdominal obesity, a well-known cardiovascular risk factor. This has already been reported in a previous study, in which a VLCKD led to a significant reduction in visceral fat assessed by DXA software and multifrequency BIA [16]. The reduction in VAT is of the utmost importance, as excess visceral fat mass provokes chronic low-grade inflammation through an imbalance in adipokine production by the adipose tissue [20]. Cunha et al. used a noninvasive, quantitative, magnetic resonance imaging technique to compare the effects of a VLCKD versus a standard LCD on VAT in patients with obesity [21]. The findings showed a greater reduction in VAT and liver fat fraction in patients undergoing the VLCKD compared with the LCD [21]. Other studies using DXA have also revealed a reduction in VAT after a VLCKD [7, 22, 23]. The study by Moreno et al. aimed to evaluate the long-term effect of a 2-month VLCKD as a treatment for obese patients [22]. The reduction in VAT, measured by DXA software, was greater in the VLCKD group than in the LCD group and this difference was still evident 2 years after the intervention. This finding, together with preservation of the FFM and skeletal bone, highlight the beneficial effect of the VLCKD as a treatment for obesity [22]. Reducing VAT will decrease chronic inflammation through a reduction in the stimulus to the secretion of proinflammatory cytokines, evidenced by a fall in serum CRP levels after the VLCKD intervention, and an increase the production of anti-inflammatory cytokines [7]. Targeting VAT will therefore decrease the risk of cardiovascular disease, diabetes, and even several kinds of cancer. Indeed, as supported by Abraham et al., CRP release from the liver correlates positively with visceral fat, typical of an obese condition, and represents a risk factor for cardiovascular events and type 2 diabetes [24].

The administration of a 3-month ketogenic diet would appear to improve the inflammatory profile, though the changes did not reach statistical significance. Specifically, we found a reduction of 0.14 mg/L in CRP values. Similar results were obtained by Merra et al. in a study conducted in overweight subjects [25]. In this case, a significant fall in CRP was observed after VLCKD, without synthetic amino acid replacement of 50% of protein intake. In contrast, Rosenbaum et al. showed that CRP increased significantly when overweight or obese patients switched from a 4-week baseline diet to a 4-week isocaloric KD [26]. Circulating concentrations of CRP, but not of the inflammatory cytokine IL-6, were significantly higher on the KD.

In the study by Watanabe et al., a 90-day dietary program consisting of a VLCKD followed by a hypocaloric LCD in obese patients, lead to statistically significant improvement of a fasting insulin and glucose, HOMA IR, and HbA1c% by the end of the study; the first phase characterized by the greatest change [27]. In the present study, the ketogenic dietary regimen led to a fall in the HOMA-IR index, although the changes did not reach statistical significance. Previous studies performed on human subjects demonstrated a decrease in insulin levels, and consequently in HOMA-IR, after a KD [28, 29]. The fall in insulin levels is related to the marked reduction in carbohydrate intake, and the main benefits of the KD probably depend on this reduction [18]. Twelve weeks of KD improved almost all anthropometric, biochemical, and hormonal parameters in a group of 14 overweight women with polycystic ovary syndrome. A significant decrease was observed in blood glucose and insulin levels, together with a significant improvement in HOMA-IR, insulin resistance was significantly decreased, falling below the HOMA-IR threshold of 2.5 [23].

A strength of this study was the assessment of two hormones: leptin and ghrelin. Leptin decreased with the VLCKD, whereas ghrelin increased; the increase in ghrelin was statistically significant. This result agrees with the study by Mohorko et al., in which a significant reduction in leptin levels was observed throughout the 12-week ketogenic diet intervention [30]. Leptin, secretion reflects stored body fat [31]. The study by Nymo et al. investigated the progressive changes in appetite and appetite-related hormones occurring during weight loss with a ketogenic diet. They observed a significant increase in basal active ghrelin in all participants, compared to baseline, only in week 13, while feelings of hunger in fasting were already reported from day 3 of the diet [32].

Interestingly, we observed a positive relationship between ghrelin and total cholesterol. This finding is in agreement with previous findings [33], further confirming the possible role of this hormone in obesity. Moreover, a similar association has also been demonstrated between leptin and insulin.

In the current study the patients were supplemented with omega-3 fatty acids (DHA and EPA). De Luis et al, demonstrated that a very low-calorie ketogenic diet supplemented with DHA was significantly superior in the anti-inflammatory effect, compared to a VLCKD without DHA, with no statistical differences in weight loss and metabolic improvement [34]. This kind of supplementation may optimize the positive effects of VLCKD on some cardiovascular risk markers and on obesity-related chronic low-grade inflammation [34]. In fact, it’s well known that the administration of DHA generates an increase in levels of intermediate anti-inflammatory metabolites that will affect the increase in resolvine among other active metabolites to solve lipo-inflammation [35]. The increase in these metabolites results in a decrease in the release of pro-inflammatory substances by these [36]. The administration of DHA manages to break the re-entry circuit between obesity and lipo-inflammation, resulting in a lower chance of regaining weight and a greater sense of satiety even in the long term.

Our study has several limitations. The most obvious is the heterogeneity of our sample with respect to age and gender. Furthermore, our conclusions should be interpreted as hypotheses that warrant further testing with larger sample sizes. As this was a pilot study, there was no control group against which to compare any changes. However, our goal was to study the effects of a VLCKD as it would be implemented in a hospital outpatient setting, which is an important strength of this study. Often, the studies on the effects of VLCKD focus only on single aspect, such as body weight [37], body composition [16, 19], inflammation [26], glucose metabolism [38] and satiety hormones [32]. The novelty of this study is the fact that it provides an overview of all these parameters. Regarding the main outcomes, several confounding factors exist. First, women were not in the same phase of the menstrual cycle for all measurements. Another strength of the study is the monitoring of the capillary ketones as an indirect measure of the fulfillment of the ketogenic diet, that has been carried out in the visits made up to the day 45.

## Conclusions

In conclusion, the administration of a VLCKD for a period of 3 months seems to be an effective strategy for promoting weight loss in patients with class I obesity. The VLCKD lead to a reduction of the body weight and the waist circumference. The FM and the VAT decreased, while the FFM showed a nonsignificant reduction. A VLCKD combined with omega-3 supplementation, therefore, is useful for promoting loss of FM with preservation of FFM in patients with class I obesity. Moreover improvements in the satiety hormones, with increased ghrelin and decreased leptin, and also in the metabolic profiles were reported.
